# Formation Mechanism of Spherical TiC in Ni-Ti-C System during Combustion Synthesis

**DOI:** 10.3390/ma10091007

**Published:** 2017-08-29

**Authors:** Guoliang Zhu, Wei Wang, Rui Wang, Chuanbao Zhao, Weitao Pan, Haijun Huang, Dafan Du, Donghong Wang, Da Shu, Anping Dong, Baode Sun, Sheng Jiang, Yilong Pu

**Affiliations:** 1 Shanghai Key Lab of Advanced High-Temperature Materials and Precision Forming, Shanghai Jiao Tong University, Dongchuan Road 800, Shanghai 200240, China; glzhu@sjtu.edu.cn (G.Z.); wei-1233@163.com (W.W.); wangrui1029@sjtu.edu.cn (R.W.); zhaochuanbao_chris@163.com (C.Z.); cornus@163.com (H.H.); dafand@sjtu.edu.cn (D.D.); wangdh2009@sjtu.edu.cn (D.W.); bdsun@sjtu.edu.cn (B.S.); 2 School of Materials Science and Engineering, Shanghai Jiao Tong University, Dongchuan Road 800, Shanghai 200240, China; panweitao123@126.com; 3 State Key Laboratory of Metal Matrix Composites, Shanghai Jiao Tong University, Dongchuan Road 800, Shanghai 200240, China; 4 Collaborative Innovation Center for Advanced Ship and Deep-Sea Exploration, Shanghai Jiao Tong University, Dongchuan Road 800, Shanghai 200240, China; 5 Jiangsu Longda Superalloy Material Co., Ltd, Wuxi 214105, China; shengjvip@163.com (S.J.); ylongpu@163.com (Y.P.)

**Keywords:** combustion synthesis, composite, TiC, Ni-Ti-C, formation mechanism, DTA

## Abstract

The formation mechanism of TiC particles in a Ni-Ti-C system were revealed by using differential thermal analysis (DTA), XRD, and SEM to identify the reaction products in different temperature ranges. The results indicated that the synthesis mechanism of TiC in Ni-Ti-C system was complex; several reactions were involved in the combustion synthesis of TiC-Ni composite. The Ni-Ti intermediate phases play important roles during the formation of TiC. Moreover, the influence of heating rate on the size range of TiC was also discussed.

## 1. Introduction

Titanium carbide ceramic materials are attractive reinforcements utilized in metal matrix composites due to a unique combination of high melting temperature (3200 °C), high hardness (3200 HV), low density (4.95 g/cm^−3^), good thermal and chemical stability, excellent wear resistance, and high fracture toughness [1]. The extremely high melting point of TiC made them essentially fabricated through powder consolidation technology. However, it is still difficult to prepare these materials from powders due to their strong covalent bonding and low self-diffusion coefficients even under high temperature and high pressure [2]. Recently, some special manufacturing technologies have been developed to consolidate them, such as self-propagating high-temperature synthesis (SHS) [3,4], reactive hot pressing (RHP), spark plasma sintering (SPS) [1,5] and transient plastic phase processing (TPPP). However, the energy- and time-intensive nature made them less practical to industry. It was also found that the ignition temperature in Ti-C mixture prepared under optimized high-energy ball milling (HEBM) conditions can be greatly decreased [6]. However, the synthesis process is complex, and further improvement is necessary for industrial application.

Compared with these special manufacturing processes, combustion synthesis [2,7] offered a technically simpler and economically more attractive route to fabricate the high-strength metal matrix composites due to a large cost-saving on the processing time, energy consumption, and high reaction purity [8]. Combustion synthesis is generally divided into two types: plane wave propagation ignited at one end of the sample and thermal explosion initiated by the uniform heating of the sample [9,10]. The latter is more attractive due to convenient operation [11]. The ignition temperature of pure TiC ceramic made by combustion synthesis from Ti and C powder blends was very high, close to the melting point of Ti (1678 °C). The introduction of suitable metal powders (such as Al, Fe, Cu) into Ti and C mixed powders is a potential solution to reduce the energy consumption and material cost of titanium carbide ceramic materials, because it can enhance the density and simultaneously lower the ignition temperature through the formation of low melting point intermetallic compounds or the liquid phase from the reverse eutectic reaction [12,13,14,15]. Ni is also a promising candidate which can greatly decrease the synthesis temperature of TiC-Ni composite and possesses a good wettability with TiC. The influence of C/Ti ratio [2] on the stoichiometry and morphology of TiC_x_ has been investigated, and the mechanism of SHS of TiC-Ni cermet was studied by means of a combustion front quenching method [16]. However, the roles of Ni-Ti intermediate phases as well as the detailed reaction procedure during the combustion synthesis of Ni-Ti-C system need to be further revealed.

In this paper, the phase composition evolution of the Ni-Ti-C system and the roles of Ni-Ti intermediate phases during combustion synthesis were studied to reveal the formation mechanism of TiC. The effect of heating rate and holding time on the microstructure of TiC-Ni composite was also investigated.

## 2. Materials and Methods

Commercial nickel powders (18 μm, 99.9% purity), titanium powders (38 μm, 99.9% purity), and carbon powders (1 μm, 99.9% purity) were used as raw materials. The mass ratio of Ni, Ti, and C in the 20 wt % Ni-Ti-C powder mixture was 20:64:16 (the molar ratio of Ti/C was 1:1). The 20 wt % Ni-Ti-C powders were mechanically stirred in a Turbula mixer for 50 min to obtain a homogeneous powder mixture, and then the powder mixture was cold-pressed into a green body. The density of the green body was about 85% of theoretical density.

The exothermic and endothermic behaviors of 20 wt % Ni-Ti-C green body were studied by differential thermal analysis (DTA), the green bodies were heated to 1200 °C at heating rates of 5 °C/min, 20 °C/min, 40 °C/min, and 80 °C/min in the DTA apparatus under argon atmosphere. To reveal the combustion synthesis mechanism of TiC-Ni composite, the green bodies were heated to different temperatures and then gas quenched; the temperature points were selected based on the DTA curve at a slow heating rate of 5 °C/min. The weight of the 20 wt % Ni-Ti-C green body used for each DTA test was just 1 g. After DTA test, the samples for microstructure analysis were cut from the sintered green bodies and then treated by grinding and polishing. The treated samples were investigated by using scanning electron microscopy (SEM, JEOL, Tokyo, Japan) together with energy dispersive spectrometry (EDS), and the phases were identified using X-ray diffraction (XRD, Philips X’pert, PANalytical B.V., Almelo, The Netherlands).

To further study the effect of high heating rate on the combustion synthesis of TiC-Ni composite, the green body of 200 g was heated to 1200 °C in a vacuum induction melting furnace and the heating rate was higher than 200 °C/min. The sample was kept at 1200 °C in the vacuum induction melting furnace for a certain holding time (15 min) in the furnace after combustion synthesis to investigate the effect of holding time on the microstructure of samples.

## 3. Results and Discussion

### 3.1. Synthesis Mechanism

**Figure 1:**
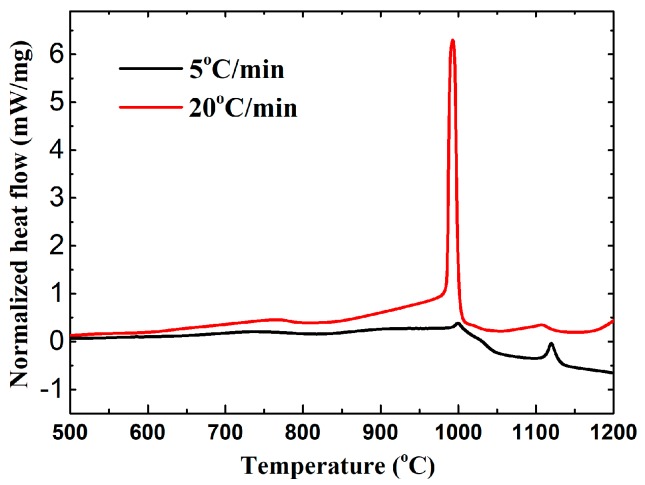
The differential thermal analysis (DTA) curves of 20 wt % Ni-Ti-C green bodies at heating rates of 5 °C/min and 20 °C/min.

**Figure 2:**
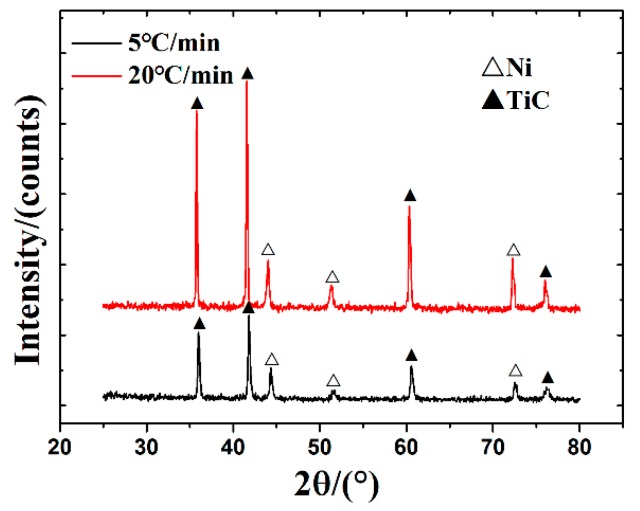
XRD patterns of the sintered green bodies after DTA tests (up to 1200 °C) at heating rates of 5 °C/min and 20 °C/min.

To investigate the combustion synthesis mechanism of 20 wt % Ni-Ti-C system, the green bodies were heated to 1200 °C at different heating rates by using a DTA instrument under argon atmosphere. The DTA curves at heating rates of 5 °C/min and 20 °C/min are shown in Figure 1. The results indicated that there were two obvious exothermic peaks and one endothermic peak in both DTA tests, and the peak positions of each DTA curve were very close, though the heating rates were different. However, the higher heating rate of 20 °C/min resulted in a much more violent exothermic reaction due to the accumulation of the reaction heat which cannot be diffused timely during DTA test, exhibiting a stronger exothermic peak than that at lower heating rate. The XRD results of 20 wt % Ni-Ti-C green bodies which were heated to 1200 °C in the DTA instrument are shown in Figure 2. The diffraction peaks indicated that the final reaction products were composed of TiC and Ni phases, though the heating rates were different, and no obvious Ni-Ti or Ni-Ti-C ternary phases were detected, which is consistent with Yang’s results [17]. It can be concluded that the reaction mechanism of the 20 wt % Ni-Ti-C system should be the same, though two different heating rates were employed.

**Figure 3:**
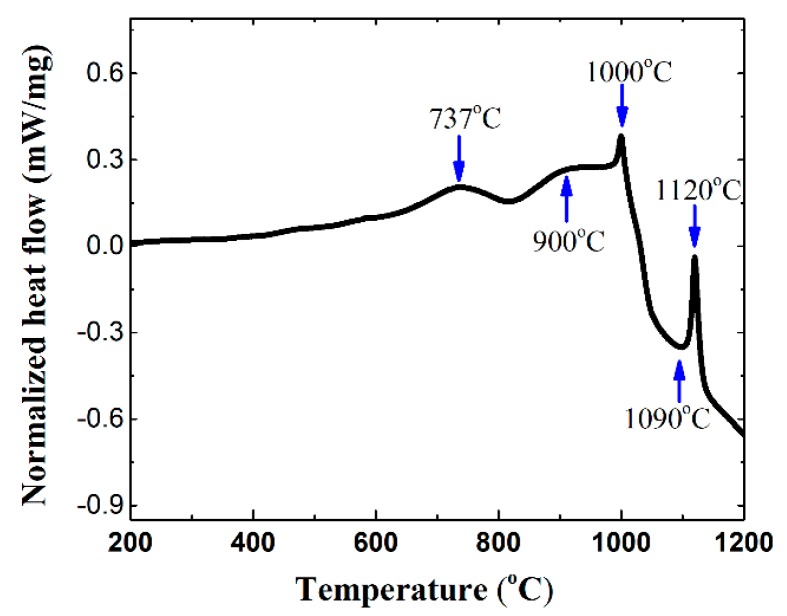
The details of DTA curve of 20 wt % Ni-Ti-C green body at a heating rate of 5 °C/min.

The DTA curve at a slow heating rate of 5 °C/min was used to analyze the change of heat flow during the combustion synthesis process, considering that the small endothermic peaks or exothermic peaks are usually covered by large peaks in the DTA curve and the details tend to be ignored when the fast heating rate is used. The exothermic peaks were found at 737 °C, 900 °C, 1000 °C, and 1120 °C, and one endothermic peak occurred at 1090 °C, as shown in Figure 3.

All the melting points of pure Ni (1684 °C), Ti (1678 °C), and C (3500 °C) are higher than 1200 °C, and the reaction temperature between Ti and C is also much higher than 1200 °C; therefore, it can be inferred that the peaks in this curve should be caused by the reaction between Ni and Ti, or the reaction of Ni-Ti-C ternary system. It is reported that TiC and Ni are the final thermodynamically stable phases of the Ni-Ti-C system in the combustion synthesis (exothermic reaction: Ti+C+xNi→TiC+xNi+Q) [2]. The quenching experiments were carried out to further reveal more details during the combustion synthesis process. The quenching temperatures were selected as 737 °C, 900 °C, 1000 °C, and 1120 °C, respectively, according to the peak positions in the DTA curve at a heating rate of 5 °C/min.

**Figure 4:**
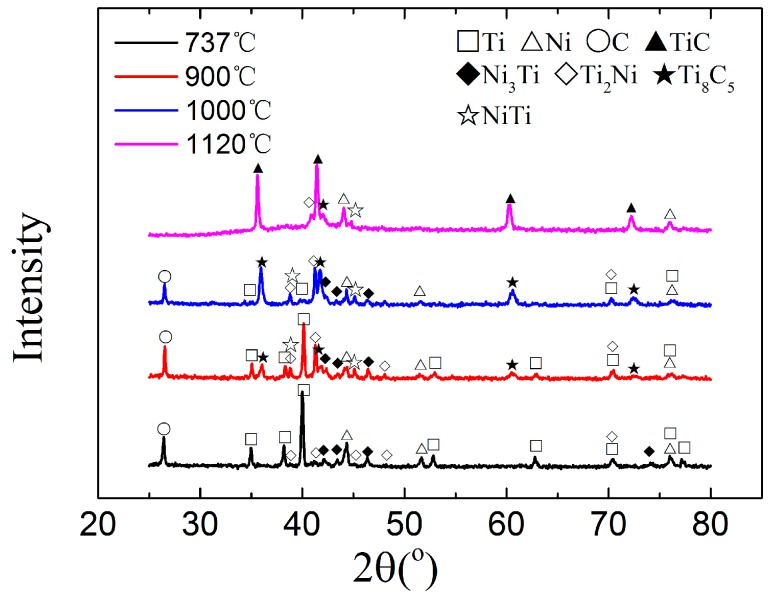
XRD patterns of sintered green bodies quenched at different temperatures with the heating rate of 5 °C/min.

Figure 4 shows the XRD patterns of the sintered green bodies which were heated to different temperatures and then quenched. The results indicated that a small amount of Ti-Ni intermetallic compounds (Ti_2_Ni and Ni_3_Ti) started to form, and the Ni, Ti, and C phases were still the main components in the sintered compact when the 20 wt % Ni-Ti-C green body was quenched at 737 °C. It can be inferred that the solid-state diffusion reaction started to occur in the contacting zone of Ti and C particles at this temperature, resulting in a locally compositional deviation of Ti or Ni [16]. Therefore, a very small amount of Ti_2_Ni and Ni_3_Ti were formed firstly, corresponding to a very mild exothermic peak in the DTA curve. The Ni-Ti intermetallic compounds were also observed in a Ti-C-25 wt %Ni reactant mixture using time-resolved X-ray diffraction [18].

The XRD pattern of the sintered green body quenched at 900 °C shows that the intensity of Ti_2_Ni and Ni_3_Ti diffraction peaks increased—especially for the Ti_2_Ni phase, which indicated that the weight ratio of Ti_2_Ni increased. Meanwhile, the content of Ni and Ti phases in the sintered green body decreased. Compared with the sintered green body quenched at 737 °C, a small amount of Ti_8_C_5_ and NiTi phases were detected in the combustion-synthesized products quenched at 900 °C. Thus, it is suggested that a few reactions took place predominantly in the temperature range of 737–900 °C. Ti reacted with Ni to form Ti_2_Ni, NiTi, and Ni_3_Ti, resulting in a significant increase of Ti-Ni intermetallic compounds. In addition, the formation of non-stoichiometric Ti_8_C_5_ could be inferred as a result of reaction between C and Ti-Ni intermetallic compounds, considering that the ignition temperature of pure TiC ceramic made by combustion synthesis from Ti and C powder blends was very high. The formation of Ti_8_C_5_ during the combustion synthesis of the Ni-Ti-C system has barely been reported in previous research work.

Compared with the XRD pattern of the combustion-synthesized products quenched at 900 °C, the synthesized products quenched at 1000 °C showed a similar phase composition; Ti_2_Ni, Ni_3_Ti, NiTi, and Ti_8_C_5_ were the main reaction products. Changes in the intensity of the diffraction peaks in XRD patterns indicated a large increase in the amount of Ti_8_C_5_, which can be attributed to a substantial formation of Ti_8_C_5_ caused by the reaction between C and Ti-Ni intermetallic compounds, benefiting from the formation of a great deal of Ni-Ti intermediate phases in the temperature range of 900–1000 °C. However, the observable intensities of diffraction peaks for Ti_2_Ni and Ni_3_Ti in the quenched samples at 900 °C and 1000 °C was similar, implying that there was a balance between the formation and consumption of Ti-Ni intermetallic compounds.

The DTA curve of the 20 wt % Ni-Ti-C system in Figure 3 also shows a sharp endothermic peak around the temperature of 1090 °C. There exists a eutectic point between Ti_2_Ni and Ti at the temperature of 942 °C according to the Ni-Ti binary phase diagram [19]. However, the composition deviation of Ni or Ti in the initial Ti-Ni liquid solution and an extremely short diffusion time contributed to a lag of eutectic temperature compared with the theoretical eutectic temperature. Thus, the endothermic peak around 1090 °C could be deduced as the endothermic transition ${\mathrm{Ti}}_{2}\mathrm{Ni}+\mathrm{Ti}\to \mathrm{Ni}-\mathrm{Ti}\;\mathrm{liquid}\;\mathrm{solution}$.

The Ni-Ti liquid solution provided an excellent channel for the diffusion and transport of elements; a Ni-Ti-C ternary liquid solution formed along with C particles dissolving into the Ni-Ti liquid phase, the TiC particles precipitated subsequently from Ni-Ti-C liquid solution as the solution was saturated [3]. Meanwhile, Ti_8_C_5_ turned into stoichiometric TiC by the convenient supplement of carbon atoms in the Ni-Ti-C liquid phase. Consequently, TiC and Ni were the predominant phases detected in the XRD pattern of sintered green body quenched at 1120 °C. A small amount of intermediate phases remained in the sintered green body after quenching test at 1120 °C, indicating an incompleteness of the combustion synthesis at this stage. There should be a small amount of pure Ti and pure C remaining in the sintered green body according to the Ti/C ratio in raw material and the reaction process described above. The diffraction peaks of pure Ti and pure C were not found in the XRD pattern due to a very small amount of Ti and C.

As the temperature continued to rise above 1120 °C, the DTA curve showed an endothermic reaction peak corresponding to the formation of Ni-Ti liquid solution from a reverse eutectic reaction between NiTi and Ni_3_Ti according to the Ni-Ti binary phase diagram (theoretical eutectic temperature is 1118 °C [20]). Then, the newly formed Ni-Ti liquid solution, the remaining Ni-Ti intermediate phases, Ti_8_C_5_, and the remaining few pure Ti and pure C finally transformed to Ni and TiC following the reactions described before.

### 3.2. Microstructure

**Figure 5:**
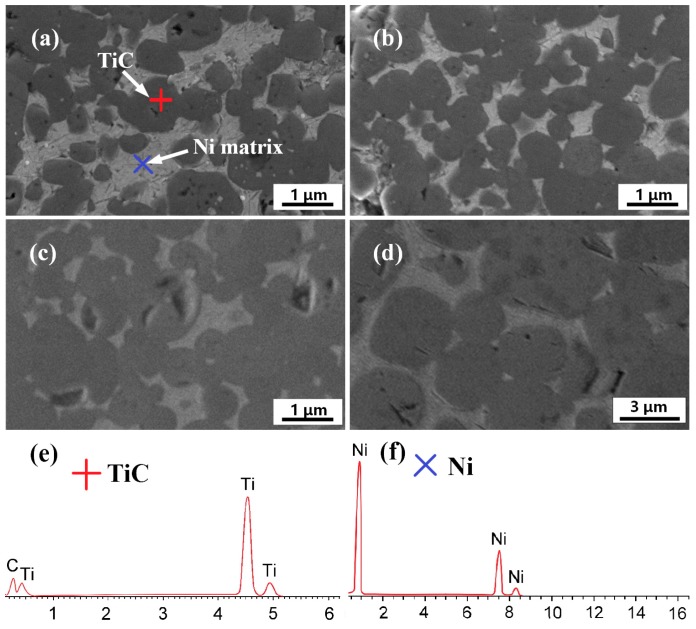
SEM micrographs of sintered green bodies in DTA instrument with different heating rates: (**a**) 5 °C/min; (**b**) 20 °C/min; (**c**) 40 °C/min; and (**d**) 80 °C/min; (**e**,**f**) The energy-dispersive spectrometry (EDS) spectra of TiC and Ni, respectively.

The TiC-20 wt %Ni composite can be prepared by combustion synthesis using a 20 wt % Ni-Ti-C powder system, and the morphology of TiC-Ni composites synthesized in DTA instrument at different heating rates are shown Figure 5. The spherical micron-scale TiC particles were uniformly distributed in a nearly continuous Ni matrix. The size of spherical TiC particles increased with the increasing of heating rate; the TiC size range varied from <1 μm to 2–5 μm when the heating rate was increased from 5 °C/min to 80 °C/min. The coarsening of TiC particles can be explained as follows: A higher heating rate led to a faster heat accumulation, including the input of an external heat source and the exothermic reaction of combustion synthesis once ignited. Simultaneously, the rapid rise in temperature resulted in a very violent reaction, resulting in a greater number of Ni-Ti liquid phases which can provide a more efficient diffusion channel for C atoms. Consequently, the growth-driving force of TiC was reduced, and the precipitation of TiC from the saturated solution and the grain coarsening of TiC particles became much easier.

**Figure 6:**
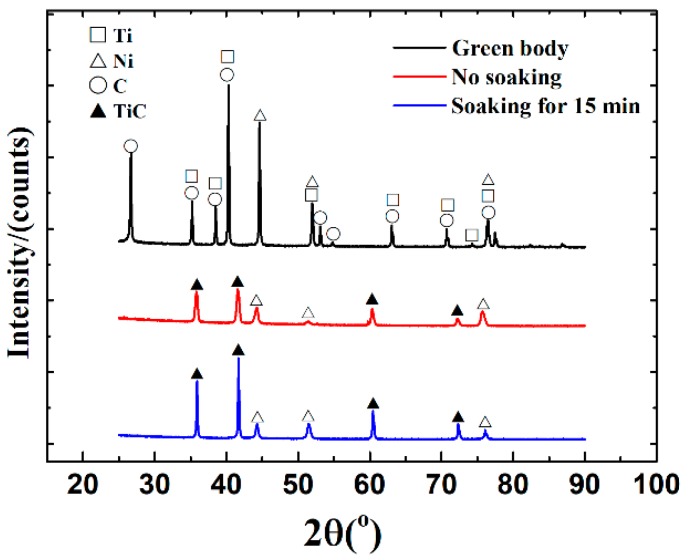
XRD patterns of the original green body, and the sintered green bodies with and without soaking for 15 min at 1200 °C in a vacuum induction melting furnace.

**Figure 7:**
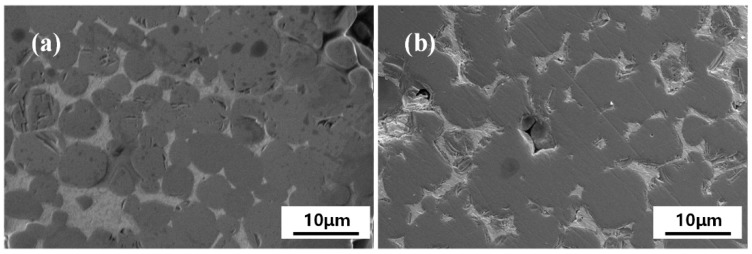
SEM micrographs of the sintered green bodies in a vacuum induction melting furnace. (**a**) No soaking and (**b**) soaking at 1200 °C for 15 min.

The larger-size samples (~200 g) were fabricated by combustion synthesis in a vacuum induction melting furnace with a heating rate (>200 °C/min) to investigate the preparation feasibility of large-size TiC-20 wt %Ni for industrial application. Figure 6 shows the XRD patterns of samples without soaking and with soaking for 15 min in vacuum induction melting furnace at 1200 °C. Phase composition analysis indicated that the combustion synthesis reaction was completed in the vacuum induction melting furnace. TiC and Ni phases were the final reaction products, and no intermetallic compounds were detected. The SEM micrographs of the sintered green bodies by using a vacuum induction melting furnace are shown in Figure 7. The size of TiC particles slightly increased after soaking. The TiC size range prepared in the DTA instrument at a heating rate of 80 °C/min was about 2–5 μm, and the size range of TiC prepared in the vacuum induction melting furnace without soaking was slightly increased to 3–10 μm. This indicated that the influence of heating rate increase on the TiC size is not significant when the heating rate is greater than 80 °C/min.

## 4. Conclusions

The TiC-20 wt %Ni composite can be prepared by combustion synthesis using a 20 wt % Ni-Ti-C powder system. The combustion synthesis mechanism could be concluded as follows: Firstly, Ti_2_Ni and Ni_3_Ti were formed through solid-state diffusion reactions between Ni and Ti at the initial stage of combustion synthesis under low temperature. In the temperature range of 737–900 °C, Ti_2_Ni, Ni_3_Ti, and NiTi were formed due to a higher rate of solid-state diffusion reactions between Ni and Ti, meanwhile the non-stoichiometric Ti_8_C_5_ was formed through the reaction between C and Ti-Ni intermetallic compounds. With a further increase in temperature, the Ni-Ti liquid phase began to form due to the reverse eutectic reaction between Ti_2_Ni and Ti, then C particles dissolved into Ni-Ti liquid solution to form Ni-Ti-C liquid solution, and TiC particles precipitated subsequently from the saturated liquid solution. Meanwhile, Ti_8_C_5_ turned into stoichiometric TiC by the convenient supplement of carbon atoms in the Ni-Ti-C liquid phase.

During the combustion synthesis of the 20 wt % Ni-Ti-C powder system, the size of spherical TiC particles increased with the increasing of heating rate; the TiC size range varied from <1 μm to 2–5 μm when the heating rate was increased from 5 °C/min to 80 °C/min. However, the influence of heating rate increase on the TiC size was not significant when the heating rate was greater than 80 °C/min.
